# “I Didn't See It as a Problem, I Thought It Was Going to Be Taken Away”: Narratives From Family Members of Users in Rehab

**DOI:** 10.3389/fpsyt.2021.649961

**Published:** 2021-08-17

**Authors:** Ariagor Manuel Almanza-Avendaño, Martha Romero Mendoza, Anel Hortensia Gomez-San Luis

**Affiliations:** ^1^Facultad de Ciencias Humanas, Universidad Autónoma de Baja California, Mexicali, Mexico; ^2^Dirección de Investigaciones Epidemiológicas y Psicosociales, Instituto Nacional de Psiquiatría, Ciudad de México, Mexico City, Mexico; ^3^Facultad de Ciencias Humanas, Universidad Autónoma de Baja California, Mexicali, Mexico

**Keywords:** substance use, family, social construction, discourse, recovery

## Abstract

There are multiple discourses on addictions that influence the way in which relatives interpret the substance use of a family member. The purpose of this study is to understand the influence of these discourses on the construction of use as a problem by relatives of people in recovery. Narratives were obtained on the path of the illness to identify the phases in the construction of use as a problem and the influence of the discourses on each phase. The process has four successive phases: normalization, impasse, exasperation, and adoption of the treatment ideology. This process goes from the legitimization of use to its moral interpretation and subsequently to the transition to medical discourse. It is concluded that it is important to reduce the influence of the moral discourse in order to facilitate timely detection and early care, as well as to design interventions focused on the reconstruction of use as a problem.

## Introduction

For health services, timely identification of substance use is important because it makes it possible to determine whether the person with substance use problems requires treatment, select the appropriate treatment, and make an early intervention to prevent use from creating serious psychosocial consequences for the person with substance use problems and those close to them. Although, the treatment process usually focuses on the person with substance use problems, incorporating family members is essential for several reasons. Firstly, use creates a burden that must be acknowledged and addressed (1). Previous studies indicate that use brings about consequences for the relatives such as uncertainty, concern for the person with substance use problems, emotional and physical discomfort, financial problems, conflicts and dilemmas associated with the management of substance use, restriction of social life, embarrassment about use and isolation, ambivalence toward the family member, decrease in the quality of family relationships, and oscillation between coping strategies (2, 3).

Although, the start of the recovery process has been linked to cathartic experiences or epiphanies by people with substance use problems, it is also promoted by family members (4). People with substance use problems seek treatment because their use causes concern and emotional distress in family members. They are forced to attend or receive the support and motivation of people close to them (5). During treatment, family members can facilitate recovery through honest feedback, instrumental and emotional support, or by becoming the motivation for change. However, they can also encourage relapses when they allow use, place demands on the person with substance use problems which they are unable to handle, or provide support during treatment (6). In order for family members to become key agents in the recovery process, it is necessary for them to construct substance use as a problem, and for the way they construct the problem to be consistent with the construction of the institutions that provide care. Previous studies have focused on the consequences of consumption on family members and their participation during the care process, but the way in which they construct the consumption problem over time has not been explored.

From a socio-constructionist paradigm, problems determine families as relational and linguistic systems. A system of the problem is constructed on the basis of the people who speak or adopt a position regarding the problem, act in relation to it, and influence the problem (7). Accordingly, there will be different perspectives on substance use in a family. These perspectives imply positions regarding the degree to which use is perceived as problematic and different strategies for its management.

The way family members construct substance use as a problem is influenced by discourses about addictions that circulate in their sociocultural context. In the trajectory spanning the process from onset of use until recovery, moral and medical discourses exert the greatest influence (8). In the present study, it is considered that the construction of the problem of consumption by family members can be affected by other complementary discourses. Likewise, it seeks to understand the way in which the influence of discourses is transformed during the course of suffering and care.

## Discourses on Addiction

Discourses are systems of statements that produce objects of knowledge and govern the way people talk, think, or act in relation to a topic, as a result of which they also influence the regulation of behavior. Discourses make certain subject positions available, since they determine how the person to whom they refer can speak or act. Discourses are also related to the interests of particular institutions occupying positions of power within society (9).

There are multiple discourses about addictions. Legal discourse distinguishes between legal and illegal substances, and contributes to their being regarded as dangerous, not only as a result of biological criteria. Illegal substance use is associated with antisocial behavior; the person with substance use problems is perceived as an agent, since he is responsible for his actions and his legal sanction is promoted. Conversely, in economic discourse, substances are regarded as merchandise, and the person with substance use problems has agency because he actively makes decisions in terms of his substance. Industry has an interest in legitimizing and normalizing use to increase profits. For its part, political discourse creates a distinction between “us” and “them.” The latter include users who deviate from social norms and are perceived as a threat. The negative representations of society are projected onto them, they entail costs in terms of health, and must be monitored (9).

One of the most influential discourses in care services is medical discourse. It assumes that substances are pathogenic and cause addiction. Addiction has been constructed as a chronic illness of the brain, since continuous repeated use modifies its structure and functioning, in addition to encouraging compulsive substance use despite the harmful consequences (10). In addition to the knowledge of the cause of the addiction and the biochemical and physiological changes responsible for the symptomatology, addiction is conceived as a disease because characteristic syndromes have been identified for each type of substance, as well as diagnostic criteria, their evolution, and prognosis (11). Recently, it has been thought that addiction is not only a process of neuroadaptation, but that there is a dual pathology, since it may be preceded by a previous psychiatric disorder (12).

Unlike previous discourses, in medical discourse, subjects are passive. It is assumed that addiction is an involuntary condition and that the subject's actions are caused by something external to them, over which they have no control (13). Addiction is constructed as a chronic, lasting disease, with expected relapses, and lifelong, only partially effective, treatment (14).

In contrast to medical discourse, there is a moral discourse, influenced by institutions such as religion and the family. Use is perceived as an evil act, either because it is illegal or because it is excessive. This discourse establishes dichotomies between use-sin and abstinence-purity. The distinction constructs two subjects: the deviant user, who becomes intoxicated and has a certain agency, meaning that he is responsible for his actions; and the person who does not use substances, and is therefore considered pure, good or virtuous. So, the person with substance use problems is conceived as a figure that represents poor decisions, loss of control, and failure to engage in essential activities such as work (15).

In moral discourse, since it is assumed that willpower is the sole or main factor in the eradication of addiction, it is not considered necessary to seek help at health services and engage in therapeutic, pharmacological, or psychosocial interventions or involve the family. Constructing addiction as a “vice” maintains the stigma that seeking health services implies publicly acknowledging oneself as a person with substance use problems, as a result of which social exclusion is feared (8).

The different discourses may intersect or complement or oppose each other. Certain discourses may become dominant and be conceived as worldviews or schemes accepted by the majority of society. These dominant discourses are internalized by individuals, as schemes that frame their perception of substance use (9). In the present study, it is assumed that during the trajectory of use and recovery, the way use is constructed as a problem can be modified, as the influence of certain discourses is expanded while that of others is reduced.

## Reconstruction of Use as a Problem

Previous studies reveal the presence of various mechanisms that can affect the way substance use is constructed as a problem. For example, care systems are designed primarily for people who recognize the need for help and are willing to receive it (16). Care policies establish the temporality of the treatment and delimit its scope, since they separate the use of other vital issues or marginalize the needs of people with substance use problems (17). Care services therefore determine the appropriate solutions for the problem of use, contribute to its being perceived as a chronic or incurable problem, and can separate use from the user's social context.

The way substance use is constructed as a problem can be modified after adopting the treatment ideologies of care services, which are unique manifestations of medical discourse in local contexts. These ideologies are specialized sets of beliefs about the nature of the substance use problems and the best practices or treatment strategies. Ideologies differ as regards whether addiction is considered an illness; the degree of influence of biological, psychological, or social factors; the emphasis on the individual or the context for recovery; the need for abstinence; and the type of treatment required to promote recovery (18).

Care services become narrative environments that express concrete expectations of change, ways of talking about change, or setting goals, and thus, shape users' narratives about their recovery (19). Iwona (20) found that people in recovery usually adopt the ideologies of the institutions where they are treated, depending on the level of exposure to treatment, and the interaction between the constructions of people in recovery and care service personnel. Internal and external people with substance use problems used to describe addiction as an incurable illness, requiring chronic treatment oriented toward abstinence, due to the inability to control use. Conversely, people with substance use problems who changed without requiring treatment considered that addiction was a sign of social malfunctioning, yet curable through willpower.

In relatives of people with substance use problems diagnosed with concurrent disorders, a process of reconstruction of the problem has been identified, beginning with the adoption of medical discourse, contact with health services and relations with other families. Use is linked to the presence of a dual pathology, the problem is conceived of as chronic and with expected relapses, and rather than treatment focusing exclusively on the patient, the need for self-care in relatives is incorporated as part of the recovery process (16).

The purpose of this study is to understand the ways family members construct substance use as a problem, through the trajectory from the start of use to recovery. It seeks to investigate how the discourses on addiction present in the sociocultural context influence the way use is problematized, as well as the role played by treatment ideologies in the reconstruction of the problem. It also attempts to identify the elements that promote the reconstruction of use as a problem throughout the trajectory in the narratives of relatives.

## Method

The study was conducted at a hospitalization and rehabilitation clinic for addictions in the municipality of Victoria, in the state of Tamaulipas, in the north of Mexico. Its model is multidisciplinary and consists of an internment program to treat acute intoxication and withdrawal, carry out a comprehensive diagnosis, and incorporate people with substance use problems into individual and family therapy; as well as the program of continuous care and reinforcement once the user has recovered. The clinic has the capacity to serve 46 people in hospital, however, on average they serve between 10 and 15 people, the vast majority men.

The rehabilitation clinic where the study was carried out is a very unique space, since there were only two public centers of this type in the entire country at the time the research was carried out. This is because quality rehabilitation centers in Mexico belong mainly to the private sector and are not available to most of the population. People with substance use problems often attend first-level centers or AA groups because they prefer outpatient treatment to hospitalization. An option available to families that do not have resources for private services are the Residential Centers for Mutual Assistance for Addiction Care (CRAMAA), also popularly known as “Annexes.” However, the degree of regulation of these care centers by the State and the professionalization of services have been questioned. Violations of the human rights of users have even been reported. Unlike the rehabilitation clinic where the study was carried out, these care alternatives do not usually incorporate family members in the treatment process or provide specific interventions for family members.

Among other reasons why the clinic does not serve a greater number of people are the limited dissemination of the clinic in the city and incidents related to organized crime, since they have been victims of attacks where they have even shot staff surveillance or the patients themselves, belonging to rival groups, have staged fights inside the clinic. Due to the activities carried out by drug traffickers in the city, and to the dispute between rival organizations, there is a high level of violence and insecurity i.e., expressed in shootings, kidnappings, extortion, and forced disappearances, among other crimes.

Participants were told the purpose of the research and given an informed consent form. Narrative interviews were conducted, which were audio-recorded and transcribed for analysis. The methodological approach used was narrative analysis, to identify the phases that family members go through from the beginning of consumption to recovery. In addition, a critical analysis of the narratives was carried out to understand the presence of discourses on addiction in each phase. The research protocol was approved by the Research Ethics Committee of the “Migration, development and human rights” Academic Group, affiliated to the Autonomous University of Tamaulipas.

Participants were purposely selected on the basis of the following criteria: being adults, being relatives of a person with substance use problems who has been diagnosed with a substance dependence disorder, and for the user to be in a recovery process. Seven family members participated in the study. The participants included six women and one man and were mainly wives or mothers. They had an age range of 23 to 51 years, and had various occupations: housewife, student, shopkeeper, employee, government official, and addictions counselor. Most were married, and only two were separated or divorced. Care service users were mainly male, and seeking treatment for poly-consumption, particularly of alcohol, hallucinogens or opiates. They had an average length of use of 11.16 years (with a range of 1 to 28 years) and an average recovery time of 5.33 years (with a range of 1 to 23 years). Family members reported one to five hospitalizations.

Two main trigger questions were used for the interviews. The first was: “Can you tell me the story since you found out that your family member was using substances until the moment he was admitted to this rehabilitation clinic?” On the basis of this question, the participant was asked to construct his story and several topics were subsequently explored: start of use; beliefs and affects about use; actions or practices before use; biopsychosocial consequences of use; indicators of aggravation of use; relationship with social support networks and care institutions.

The second question for triggering narratives was: “Can you tell me the story of your relative's recovery, from the beginning of the recovery to the present?” In order to support the construction of the story, the following topics were explored: hospitalizations; reasons for hospitalizations; practices for encouraging hospitalization; beliefs and affects about recovery; the relative's participation in treatment; and the changes achieved through treatment.

Interviews lasted between 1 and 3 h and were done by one of the researchers. These interviews were realized with most of the relatives of people with substance use problems that attended the center in the moment of the study; only two persons refuse to participate.

Interviews were analyzed by two researchers. Analysis process was made in three phases. In the beginning, a narrative analysis was performed where the thematic focus was to identify the plot development and its dynamic was interpreted according to the participant evaluations and reflections, also with the use of terms that refer change or stability. Afterwards stories were compared to identify recurrent patterns, common themes and divergences in the narrative forms (21).

This first analysis phase allowed to identify those ones were relatives passed from the beginning of consumption to the actual treatment moment. According to the premise that families are systems that construct problems through language (22–24), it was considered that relatives constructed the consumption problem through the phases and this construction was manifested in their saying and feelings toward consumption, the relationship they stablished with the person with substance use problems and the strategies used to manage consumption.

In a second phase, a narrative categorical analysis was made (21), assisted by the software MAXQDA 12 (25). The purpose of this analysis was to identify categories that permit comprehend the particular conditions of each phase. Codes were generated inductively from data to identify beliefs, affections, practices, relationships with the person with substance use problems and with external agents of the family, in order to stablish differences among phases. It was made an open, selective, axial and theorical coding (26), that gave account of the recovery process and the conditions that permit transition among phases.

The last analysis phase started with the premise that narratives are molded by dominant discourses, which stablish forms or parameters that affect the way people perceive themselves and restrict their action possibilities (27). Dominant discourses toward substance consumption restrict the way persons interpret the consumption of the person with substance use problems, understand the consumption management and the way they connect with their relatives and also with care centers.

It was performed a critical analysis of narratives (28) to identify the influence of these dominant or canonical social discourses in the interpretation of consumption as a problem. It was assumed that meaning give to consumption are affected by a discourses polyphony, but that there is a certain agency degree of relatives to question them and negotiate replacement discourses that enable them to adopt alternative positions as subjects. To promote the trustworthiness of the findings, an audit trail of the analysis process was developed and a triangulation of the analysis was performed by two of the researchers. After carrying out the analyzes independently, meetings were held to contrast the identified phases, the characteristic processes of each phase and the influence of the discourses on addictions.

## Results

Four phases were identified in the process of the construction of substance use as a problem by relatives. In each phase, various discourses on addictions converge, although, there are phases in which one of them becomes dominant. In addition to the influence of discourses, changes in the construction of use are associated with the deterioration caused by use, affective changes, and the relationship with the person with substance use problems, as well as the link with care services.

### Normalization

During the first phase, substance use was not perceived as problematic behavior, but considered normal behavior. Normalization occurs in sociocultural contexts where use is accepted as a ritual of socialization in everyday life. Two elements that modify the normalization of use were identified. One is the legal status of the substance because the use of legal substances such as alcohol is legitimized, whereas, the use of illegal substances such as cocaine or marijuana is sanctioned to a greater extent. The second element is gender regulations of use, due to the broader acceptance of use by men than women.

Characteristics of the person with substance use problems that contributed to normalization were identified. For example, early onset of use meant that the behavior was perceived as a habit formed in the long-term and as part of a lifestyle. The condition that may have played the greatest role in normalization was the maintenance of functionality by the person with substance use problems, in areas such as the work context, education, and home:

*Right now, I recognize that he is what they call a productive alcoholic… in fact, that's why it has been difficult for me… to separate from him, because he is an excellent provider* (woman 1, wife).

Normalization was also linked to the ability of people with substance use problems to hide their use from family members. Concealment involved the reduction of the number of relatives who know about the person's use, covering up episodes of use to alter the perception family members have about its frequency or severity; and not revealing the type of substance used. This was particularly true of illegal substances, since they affect the way people with substance use problems are perceived by others due to their association with dangerousness or moral deviation:

*And of course, I realized but she lied to me, in other words, I used to take her to parties and say: hey,… you reek of tobacco… I can feel the smell here, in my throat… but she always denied it… and it was as though I wanted her to deny it* (woman 4, mother).

During this phase, relatives perceived use as a normal situation rather than a problem requiring action from the family or outside the latter to solve it:

*First of all I did not regard it as a problem… I thought he was going to get over it, that he was going to say, “Well, I'm married now”, I thought, ‘I'm married, I have a wife now, I have to work, I can't get drunk, I can't drink” – those were my thoughts* (woman 3, wife).

It may be that during the normalization phase, relatives did not perceive that the patient had an addiction, and therefore, the medical discourse of the addiction as an “illness” was absent. Use was not considered problematic in legal discourse either, because it was initially restricted to legal substances or, because the use of illegal substances, sanctioned by this discourse, was concealed. In a context where the use of legal substances is normalized, there is a predominance of economic discourse, given that the substance is considered a commodity and the person with substance use problems is an agent who chooses to use it in a rational manner.

The moral discourse of addiction was not fully expressed in this phase either because it was not conceived as a negative or immoral habit and the person with substance use problems or relatives were not stigmatized. However, two elements of that discourse were present: it was assumed that use was voluntary and that the person with substance use problems was responsible for reducing its use.

### Impasse

There was a gradual increase in use that led to a questioning or breakdown of normalization among the relatives of the person with substance use problems. This exacerbation was characterized by an increase in the frequency or dose of use; an alternation between episodes in which everyday life is “normal” and episodes in which it ceased to be “normal.” There was a psychosocial deterioration characterized by the lack of control over use, the beginning of poly-consumption, psychological damage, and a decrease in functionality, which was expressed in school and work problems, criminal behavior, or moving to a new house. In contrast to the previous phase, spheres of everyday life that could not be normalized were detected, and there was a loss of the functionality that had previously offset use and the obvious psychosocial deterioration of the person with substance use problems:

*Well, it was always normal for them to have some drinks, but this kept increasing. And before it was weekends, and then it was midweek, it did not matter whether it was Monday, Tuesday, Wednesday… it fluctuated between what was normal and what was abnormal* (woman 1, wife).

In their stories, participants used various phrases to describe their lives during this phase: “horrible years,” “experiencing madness,” “subsisting,” or “normalizing the abnormal.” This phase can be affectively characterized by the ambivalence experienced by relatives: oscillating between living and “being consumed”; worrying about the person with substance use problems and distancing themselves from them; feeling the desire to leave them and being unable to do so; experiencing a conflict between helping and not helping; and showing compassion and at the same time anger. During this phase, relatives experienced an unresolved dilemma, and remained paralyzed, even though, they recognized that the situation had stopped being normal and they felt uncomfortable as a result of the degree of their relative's use:

*I spent my whole time in a situation where I did not know what to do… whether or not to help him* (woman 5, mother).

During the impasse, relatives hoped the person would stop using substances, which they expressed through phrases such as, “he'll get over it,” “he'll stop using because of the family,” “I am going to change him,” or “he'll get better without having to get treatment.” This position overestimated the ability of the person with substance use problems to change on their own, without seeking health services specializing in addiction or with the support of the family alone:

*I remember that he was in my parents' room and he began to talk to each of us as though he was asking for forgiveness, and to explain and promise that it would not happen again, that he was with us, that he loved us very much, my mom, and at the end, he talked to us all again and we hugged each other and it seemed everything was fine* (woman 2, daughter).

As happened during the previous phase, during the impasse, use was not conceived of from the perspective of medical discourse. However, use began to be constructed as a problem for the relative and various elements of moral discourse are present in this construction. First of all, they began to perceive that use was a negative habit. The image of the person with substance use problems began to deteriorate, since he began to regard himself as “irresponsible,” “unreliable” or “weak” as regards the substance. Use was perceived as a voluntary act, so the person was expected to be able to change through willpower, without having to seek care services or another form of external support. Lastly, the relative thought that use was a problem whose solution depended exclusively on the person with substance use problems, even though, they suffered the consequences of this use in everyday life. During this phase, medical discourse was not expressed and the person with substance use problems was not thought to require specialized treatment for addictions.

### Exasperation

The exacerbation of use and the impasse could have extended over time until the moment when relatives recognized that the situation had reached a limit and that action was required for its resolution; in other words, a decision was made to stop the person's use. This phase can be regarded as the moment when the family member “hits rock bottom,” which did not usually coincide with the time when the person with substance use problems “hits rock bottom.” This means that the relative can modify their position toward the problem and the strategies for its management, but this does not usually stop use immediately. It is often followed by resistance to treatment, and cycles of hospitalization and relapses by the person with substance use problems:

*She came to the house and kept on dialing my number and I did not pick up the phone until the woman who cleans the house rang me. She said, “Here's L., but I said you told me not to let her in… she's outside but she's crying her eyes out and says she wants to talk to you”. I said, No, I do not want to talk to her' and I did not answer her. I said, “Let her be, let her sit out there on the bench, in the sun”. Then I plucked up my courage… what I really wanted was to hospitalize her. Later on, the cleaning lady rang me again and said, “She says she wants to be hospitalized”* (woman 4, mother).

The relative's exasperation was prompted by the presence of events or critical events linked to substance use. Different types of events were identified in the accounts: problems with the law, injuries or accidents, stopping work or dropping out of school, disappearing for days, a relapse in use despite going to treatment, acceptance of the disease by the family member, or even becoming aware of the use which had previously been concealed:

*So, he left us a message saying that he was fed up with us, that he wants to lead his own life and who knows what and he took the TV screen with him. He went and sold it and left for (city)… a few days later he talked to my wife and told her he was very sorry for having done that… and so on and so forth… and asked her to send him money so he could come back… So, I said to my wife, “Let him come back on his own, because that's how he left, let's see what he manages to do”* (man 7, father).

Something that indicates that the relative has “reached rock bottom” is a change in the way they identify the person with substance use problems. The positive identification that remained during the normalization phase has become a negative identification. Another element was the recognition of the lack of control of the people with substance use problems over their own use and of the ineffective attempts made by relatives to change their behavior. The relative faced the dilemma between allowing consumption or demanding to stop.

*Until one day I saw the light. My son continued to use, he had come back from [the city] again, and gone from hospitalization to hospitalization … and why should I allow him to stay at home, using? Because the program was telling me, it does not depend on you… it depends on him… but in my heart of hearts, I said, “I have to do something”… I cannot leave him like that, because I had already allowed him to take drugs at home, and to be there all the time, and not to leave, because I was worried something might happen to him in that state* (woman 5, mother).

When the dilemma was resolved in favor of stopping use, it was assumed that the person with substance use problems could not stop using on a voluntary basis and required the support of specialized services given that use had become a persistent and irreversible “vice.” Moral discourse continued to predominate in this phase, but a reconstruction occurred: it was recognized that the person with substance use problems no longer had control over use and required external treatment. That is why, during this phase, seeking treatment was encouraged and limits were established for the person with substance use problems to go in for treatment:

*He came here of his own accord… not with lies, we did not bring him in… or tie him up or anything… he signed …of his own volition… but knowing… that… if you are not going to help… well, I'll go to my daughter's* (woman 6, wife).

In this phase of exasperation, ambivalence decreased and negative affectivity increased. Participants expressed feelings of anger, resentment, distrust, or embarrassment toward the person with substance use problems. This type of affectivity signals the imminence of exasperation and encourages the mobilization of relatives to adopt new strategies to stop use. Although, the moral discourse has predominantly influenced the construction of the problem from the impasse phase, during the exasperation stage there is a transformation that establishes the basis for the subsequent adoption of medical discourse: the problem cannot be controlled voluntarily and its solution requires specialized care.

The impasse stage sees the start of a process that becomes more acute during the exasperation stage, because the psychosocial deterioration is so severe that it alters the identity of the person with substance use problems and the family's affective bond. Political discourse is implicitly expressed since the person with substance use problems becomes a threatening figure and is identified as a deviant, whose control escapes the family network. Going to specialized care services in this phase not only represents the search for rehabilitation, but also the provision of surveillance and control at a time when the family has identified its own limits on care.

### Adopting the Treatment Ideology

The beginning of the treatment by the person with substance use problems and the interaction of family members with the care institution caused another change in the way they perceived use. First of all, relatives began to interpret the problem of use as a disease rather than a vice. This assumes that the behavior of people with substance use problems did not occur as a result of a moral failure or depend on voluntary control, but rather that their behavior was due to an illness, which is more psychological than biological from the point of view of the relatives. At the same time, there was a change in the way the patient was identified: he went from being a person with a moral problem to a patient with a health problem i.e., a recovering person with substance use problems:

*The addiction is in you, alcohol is alcohol and it does not hurt you, marijuana is marijuana, it is a plant, pills… if they are medical they are for a treatment, but they hurt you, it hurts you when they enter your body, so the illness is in you, what is illness? Obsession* (woman 3, wife).

*In fact, you find out later that what they have is… um… is not a vice… it is an illness that is killing them, unfortunately* (man 7, father).

During the treatment process, female relatives, whether wives or mothers who were more affectively involved with the patient, were more concerned with stopping use or were given more responsibility for its management; they were identified by the institutions as co-dependents. This implied that they also had an illness of a psychological nature. This diagnostic label sends the message to the relative that their actions contribute to maintaining the problem of use, and accordingly, a change is encouraged: from blaming the person with substance use problems for his use to the shared responsibility for stopping use. This means that not only does the person with substance use problems has to modify his behavior, but the family member also needs to modify the way they interact with them:

*This is when I was told that I had an illness called co-dependency because… in fact my mother lives somewhere else, about 40 kilometers… from the city… I did not even go away for two days, because I thought that if I left… something bad would happen to her. I felt like her amulet* (woman 4, mother).

The ideology of the treatment persisted in the follow-up and influenced the adoption of a new lifestyle. This style is based on the conception of the illness as something i.e., interminable, because it is mainly of a psychological nature, and was described as an obsession that can be directed not only toward substances, but toward other objects or activities. Thus, treatment became permanent and a necessary condition for maintaining a healthy life because the risk of a relapse was always latent. Treatment is not only for the person with substance use problems diagnosed with a substance dependence disorder, but also includes the family member who has been identified as co-dependent:

*I don't find it difficult to go to my group on Saturdays, at first if, it was like… difficult, I went because I felt obliged to… I knew it was for my own good, right now I see it as part of my life, like eating, sleeping, going to the bathroom, or… it's something I need to live and I'm not going to stop going* (woman 1, wife).

During this phase, the affectivity of relatives changed from anger and exasperation to a form of “tough love,” where affection and concern for the person with substance use problems expressed itself in setting limits, the continuity of treatment, and the promotion of hospitalization following relapses, as well as participation in the treatment process during and after hospitalization.

*The family has to set limits, that is… it has already set them… but it has to maintain that limit… that is, not to move… but the limit is for me… it is, for me, so to speak. Maintaining… trying to see what is the… for example; in my case, you see? In this home you are not allowed to use alcohol or drugs, you see? As long as you live in this house, it is not allowed. So… it is hard but it can be done… and they know. Because my daughter knows, that is, she says… I see you being firm and I'm scared of you, right?* (woman 4, mother).

The adoption of the ideology of treatment is not immediate, it may require the pilgrimage of going from institution to institution, as well as a succession of hospitalizations and relapses. This ideology can be adopted when the person with substance use problems has stabilized and shown signs of improvement, such as abstinence and a return to social functioning. In this phase, it is possible to identify the dominance of medical-psychiatric discourse since use is constructed as a disease or addiction, whose origin is mainly perceived as psychological. Its solution does not depend on voluntary control but rather on long-term psychosocial treatment and the adoption of a new lifestyle, given the latent risk of relapse. In this ideology, the relative also has the illness of co-dependence, and therefore, requires treatment and needs to assume responsibility for the way they respond to the person with substance use problems.

**Figure 1:**
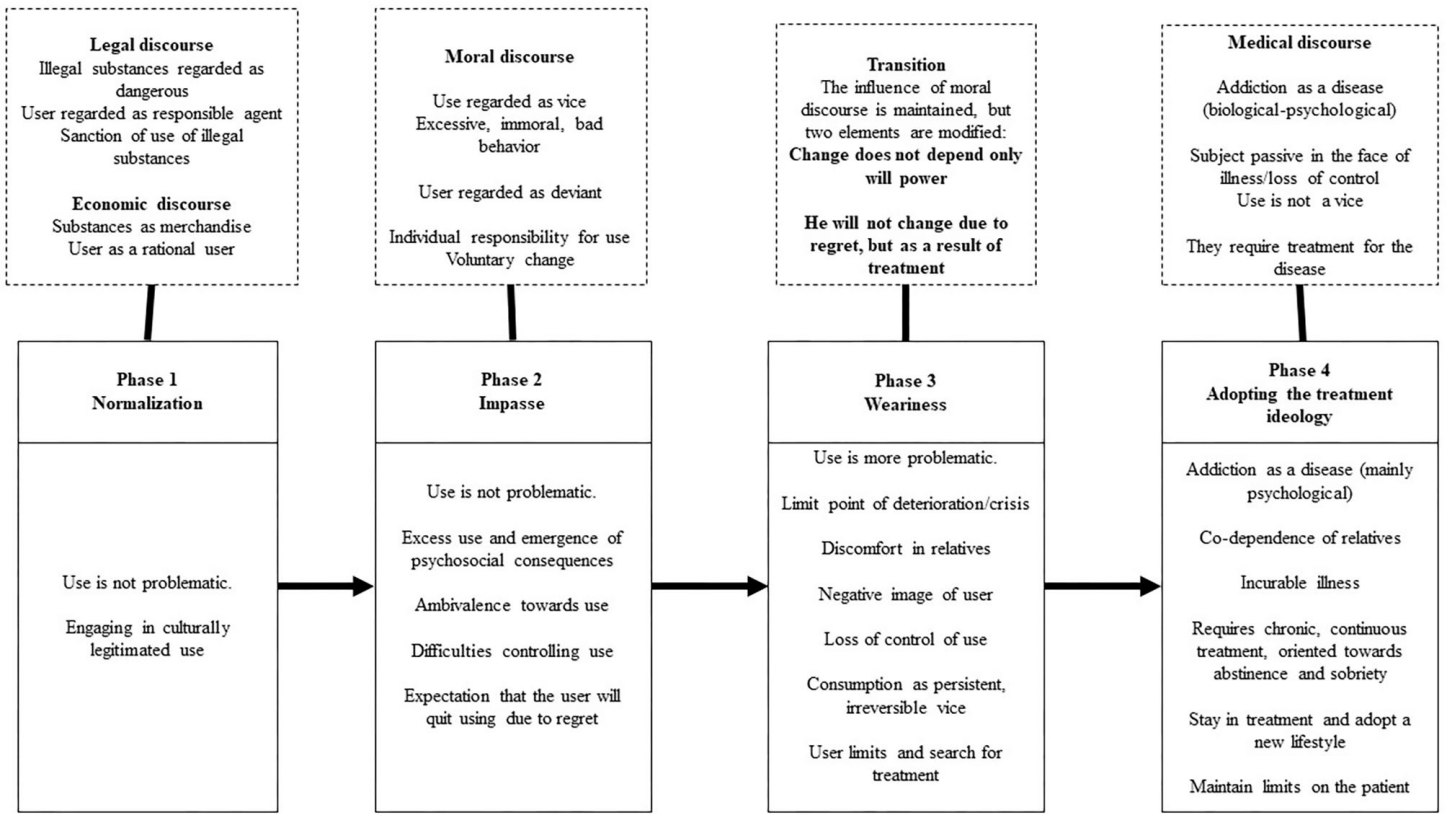
Phases in the construction of use as a problem by family members.

This phase sees a significant decrease in the influence of moral discourse since use is no longer perceived as a vice that can voluntarily be changed. However, there is a new transformation of the discourse due to the moral implications of the recovery process. Both the person with substance use problems and family members are considered responsible for remaining in recovery from the disease, and are given the opportunity to build an identity as recovering people with substance use problems if they manage to follow a new lifestyle, continue in treatment, and avoid relapses. The process of constructing substance use as a problem is shown in Figure 1.

## Discussion

From the perspective of a dynamic model of relapses, there is an interaction among several factors during a situation where there is a risk of substance use. In addition to proximal factors such as abstinence symptoms, there are distal factors like the family history and social support (29). Recovery is an interactive and context-bound process (30), in which the family can play a critical role. Substance use does not necessarily imply a problem for the members of the family, but it does need to be constructed as a problem in order to take action. The way this problem is constructed not only affects the perception family members have of the person with substance use problems, but also the practices they adopt to solve the problem. This has a major implication for practice: recovery is not only encouraged by new practices, ways of relating to or affectivity toward the person with substance use problems, it is also facilitated by a change in the problematization of use.

During the initial phase, called normalization, the substance is perceived as a commodity and the person with substance use problems as a rational agent who voluntarily regulates its use. This normalization is promoted by the intersection of economic discourse with legal discourse, since it legitimizes the use of legal substances such as alcohol, which is also usually the onset substance (9). The level of acceptance of use is linked to gender regulations, although, the use of any illegal substance must be concealed in order to maintain normalization. At this point, use has not become a moral issue, since the person with substance use problems manages to maintain their social and economic functioning in everyday life. Yusay and Canoy (31) found that young people had become accustomed to the use of substances by their parents and it had become an accepted practice in the family. A large number of families of substance users may remain in this phase and use may never become a problem. A barrier that has been previously identified is the lack of understanding of mental health problems and addictions (32), as it limits timely contact with health services. Preventive strategies should address the detection of the use of illegal substances by family members and the identification of early signs of aggravation of use. The study comprised family members, friends of the user or school staff who initially detected the use, as a result of which it is necessary to incorporate other means of detection, such as health services or work organizations.

The permanence of family members during the normalization phase may be related to processes developed by the person with substance use problems, such as self-deceit, which permit the denial of the consequences of consumption, or the manipulation of impressions to avoid a negative evaluation (33). These processes tend to reduce as the treatment advances, and so they may be used before attending care services. Likewise, it has been found out that people with substance use problems with a high social desirability have difficulties to acknowledge the need for help and tend to sub-report the severity of their consumption (34). These processes do not only limit the contact of the person with substance use problems with care services and the start of treatment, but they may also prevent family members from making an early detection of the use problem.

Rogers et al. (35) found that people with substance use problems may face difficulties in recognizing the use problem when they assume themselves as functional and adjust their consumption to stop it from obstructing their activities, mainly work-related ones. It should be noted here that the functionality notion prioritizes the labor or economic aspects over the family environment functions. Other contributing factors are stereotypes about people with substance use problems and so they look to avoid labeling. Starting treatment implies admitting one has a problem, and this is perceived as a defeat or failure (36). According to the findings, both the person with substance use problems and the family develop normalization processes about consumption which stop it from being perceived as a problem. At the start, the consequences of consumption might go unnoticed, denied, or hidden. When these is no longer possible, the handling of the image due to the internalization of the stigma becomes an element which obstructs the timely contact with the care services.

Use may be prolonged, until the point when it begins to be constructed as a problem requiring a solution. Use becomes a problem not only when there is a significant increase, but also when the psychosocial damage to the person with substance use problems becomes obvious, there is a decrease in social functioning in spheres such as school or work, and family relationships are transformed. Another element for understanding the problematization of use is affectivity. Family members go from the peace of mind and a lack of a sense of urgency characteristic of the normalization phase to concern for the person with substance use problems and a negative perspective of use.

When the family is not in touch with care services and economic and legal discourses no longer suffice to understand a type of use that has ceased to be legitimate, moral discourse is adopted as a cultural device for interpreting a type of use that has become excessive and creates discomfort in the relational context of the person with substance use problems. Within this discourse, use is interpreted as a vice, whose responsibility is individual, and change requires the will of the person with substance use problems rather than specialized treatment. As noted by Nuño-Gutiérrez et al. (8), this construction limits timely attention because the person with substance use problems is expected to change on his own, without the need for external intervention. Relatives experience ambivalence toward people with substance use problems because they care about them and at the same time they are annoyed by their use. In everyday life, they handle the consequences of use and at the same time seek to preserve the relationship with the person with substance use problems because they continue hoping they will be able to change as a result of their own decision.

The impasse may be prolonged over time as long as the family decides to address the constructed problem of use within the limits of the family context, without linking it to specialized care services. During this phase, family members may turn to those close to them, religious institutions, or even individual therapy to receive support and information. One of the practical implications of this phase is the need to create interventions to reduce the stigma toward people with substance use problems, since this situation limits the willingness of family members to talk about the problem of use with their close networks (32). This favors its construction as a domestic problem that must be addressed privately (31). It is also important for services specializing in addictions to have a greater presence in communities, both through information campaigns and networks with multiple institutions, that facilitate timely detection and referral. One condition that affects the start of treatment by people with substance use problems and their families is the shortage of health services specializing in addiction and the lack of supervision of the quality of services provided by non-governmental organizations.

The transition to the phase called exasperation arises in response to a life crisis or the exacerbation of the psychosocial deterioration of the person with substance use problems. Affectivity plays a fundamental role in this transition, since the relative shifts from ambivalence to the predominance of discomfort. It modifies the image they have of the people with substance use problems and the relationship they maintain with them. It has been reported that the expression of anger and pain by family members is an affective form of resistance, it allows to communicate disappointment because the person with substance use problems does not fulfill their obligations and justify the decision to distance themselves emotionally to avoid being hurt (31).

Accordingly, this phase is characterized by the fact that the relative “hits rock bottom,” both because of the presence of external events and the transformation of the way they want to relate to the person with substance use problems. Although, moral discourse continues to predominate in the interpretation of use, a modification occurs that will allow the subsequent adoption of medical discourse: the person with substance use problems has lost control with regard to the substance and requires professional help. It seems that family members engage in both empathic and defensive involvement (30), since they understand that the person with substance use problems suffers and requires help, but at the same time they are convinced that they need to establish limits to stop consumption and force them to attend treatment. Family involvement at this phase is crucial since a barrier for the start of treatment is the perception of a lack of social support, together with treatment costs (36). Actions are therefore needed to increase the visibility of these services and to supervise the quality of care centers, since families have to pilgrimage and go from institution to institution to find appropriate centers, where the person with substance use problems is treated with dignity, interventions are carried out effectively, and the family is incorporated into the treatment process.

At internment centers, it has been found that as the treatment progresses, the person with substance use problems develops a higher internal control (37). Still, it is the interaction between the personal internal control and the external controls of the institution that reduce the level of substance use during recovery (38). The findings of the study suggest that at the exasperation phase people with substance use problems may benefit from the external controls promoted by relatives at a time of the trajectory when they have not developed enough internal controls. Again, it should be noted that resorting to coercion on the part of the family to promote the internment of the person with substance use problems may contribute to a resistance to treatment. Retention may be eased out when the family becomes an important source of external motivation for the person with substance use problems, who in turn displays an interpersonal search for help (39).

During the last phase, there is a radical transformation of the construction of use as a problem since medical discourse is adopted, as a result of which substance use is interpreted as a disease. It should be noted that the adoption of this discourse is mediated by the institution where they seek treatment, because its ideology of care is internalized (18, 20), especially when they have been exposed to treatment for a sufficient time, changes are observed in the person with substance use problems and they continue to be in a process of recovery. In the study, the care ideology constructed use as a disease of primarily psychological origin, over which the people with substance use problems have no control and for which they are not responsible. As it is considered an incurable condition, the person with substance use problems needs to remain in chronic treatment whose goal is abstinence, which implies a change in lifestyle and avoiding the latent risk of relapse. It should be noted that although care ideologies may promote change in people with substance use problems or their families, it may limit the treatment of people with substance use problems who seek to reduce their use rather than having abstinence as a goal, as well as those whose contextual conditions or individual resources affect the possibility of adopting a new lifestyle and maintaining long-term abstinence.

Resistance to treatment is a predictor of substance use. Resistance is produced by skepticism or reject toward aspects of the treatment, the denial of the problem, or the way in which the canalization is carried out, especially if it is coercive (40). Adopting the care ideology by the person with substance use problems and the family is an element which can help to reduce the resistance toward the treatment and recovery as well. A negative perception of the treatment in the culture, being valued as not so effective or distant from their everyday experience, or having conflicts in terms of the goals, as is the case of people with substance use problems who attempt to control consumption without being abstinent themselves are among the barriers reported (36). It has also been found that during the recovery process, change is facilitated by the perceived benefits from treatment, the evaluation of the therapeutic context, and the affective involvement with the therapeutic community (41).

Although, during this phase, moral discourse has yielded to medical discourse, treatment gives people with substance use problems the opportunity to reconstruct their identity as a “recovering person with substance use problems” which entails becoming a “good patient” or a person who has “given up vice.” Attending treatment has been associated with social desirability because the person with substance use problems tries to construct a positive image of himself and show self-confidence (34).

While people with substance use problems are not considered responsible for their illness, they are regarded as being responsible for maintaining abstinence and avoiding relapses. If this is achieved, they will have the possibility of restoring their links with their relatives and restoring their reputation within the family. In the recovery process, addiction is also constructed as a problem to overcome, and agency is promoted in people with substance use problems to decide if they want to continue substance use (42). This coincides with previous findings about an increase in the locus of internal control according to the duration of treatment given that self-regulation processes are produced (37). Similarly, changes in locus of internal control locus are associated with an increase in the self-esteem of the person with substance use problems as the recovery progresses (43).

During this last phase, relatives move from concern and ambivalence toward the development of a sense of hope, supporting the person with substance use problems during treatment and focusing on self-care (16). Recovery goes from focusing exclusively on the person with substance use problems, to including family members, so that they modify the way they interact with them and attend to their own needs.

However, family members are identified as co-dependents and continue to be treated in parallel with the person with substance use problems. Harkness and Cotrell (44) have criticized the fact that this diagnostic label is mainly assigned to women, who are subordinated in substance dependence treatment and are blamed for assuming a social role previously considered normative and functional. Orford et al. (45) propose that rather than “pathologizing” family members, substance use should be regarded as a situation of chronic stress, which can be addressed through both effective and ineffective strategies. Accordingly, it is suggested that the development of effective interventions with family members be promoted, which does not imply their “pathologization,” and focuses instead on the reconstruction of use as a problem, the type of affectivity in response to use, the obtainment of information and contact with specialized services, as well as the acquisition of strategies to interact with people with substance use problems and cope with relapses.

The narratives about use and recovery constitute an adequate methodological approach for exploring changes in the construction of use as a problem over time, although, they are limited by the influence of the current dominant discourse in the narration of the past. Likewise, the findings mainly represent the perspective of mothers and wives on substance use since they usually assume a central role in the care of the user. It is important to incorporate the views of men and members with other roles in the family, and even to analyze the presence of opposing positions found in each phase on the part of members. Finally, recovery is a process that involves relapses, so it is suggested that changes in the problematization of use during treatment be explored in greater depth. For future studies, the development of ethnographic studies or participatory-action research is recommended as it will allow a closer monitoring of families during the recovery process and make it possible to identify new phases in the construction of use as a problem.

## Conclusion

Substance use is not a problem in itself for the relatives of people with substance use problems but is constructed as a problem through the trajectory that comprises the period from the start of use to recovery. During the initial phase, use is normalized, since it is interpreted on the basis of economic and legal discourses. Use is subsequently interpreted as a vice from the perspective of moral discourse, and this change is associated with increased use, the deterioration of the person with substance use problems, family ambivalence, and the alteration of the relationship with the person with substance use problems. The relative reaches a critical point in the phase of exasperation, because they realize that the problem does not depend on the will of the person with substance use problems and requires specialized support. In the last phase, recovery is facilitated by the adoption of the institution's care ideology, where use is constructed as a chronic, incurable, psychological illness that requires a change in lifestyle oriented toward abstinence and the prevention of relapses. Interventions must be adjusted to the stage in which the family is located. During the initial phases, families must identify the use of illegal substances and recognize the indicators of psychosocial deterioration. Timely access to health services involves the creation of strategies for the reduction of stigma, improvement of service quality and greater visibility in the community. The treatment process requires incorporating the family to promote the maintenance of care, as well as designing interventions focused on the reconstruction of use as a problem, the affectivity associated with use, the search for information and institutional networks, and the acquisition of strategies to interact with the person with substance use problems and cope with the difficulties associated with substance use.

## Data Availability Statement

The datasets presented in this article are not readily available because Confidentiality. Requests to access the datasets should be directed to Ariagor Manuel Almanza-Avendaño, almanzaa@uabc.edu.mx.

## Ethics Statement

The studies involving human participants were reviewed and approved by the research protocol was approved by the Research Ethics Committee of the Migration, development and human rights Academic Group, affiliated to the Autonomous University of Tamaulipas. The patients/participants provided their written informed consent to participate in this study.

## Author Contributions

AA-A: first authorship, elaborated the project, design analysis, supervised fieldwork, and wrote the paper. MR: collaborated in the discussion and analysis of the results and reviewed the final version of the paper. AG-SL: realized field work, collaborated equally in the analysis, and writing of the paper. All authors contributed to the article and approved the submitted version.

## Conflict of Interest

The authors declare that the research was conducted in the absence of any commercial or financial relationships that could be construed as a potential conflict of interest.

## Publisher's Note

All claims expressed in this article are solely those of the authors and do not necessarily represent those of their affiliated organizations, or those of the publisher, the editors and the reviewers. Any product that may be evaluated in this article, or claim that may be made by its manufacturer, is not guaranteed or endorsed by the publisher.
